# Bisphosphonate-Induced Orbital Cellulitis in a Patient With Suspected Parathyroid Carcinoma: A Case Report

**DOI:** 10.7759/cureus.63577

**Published:** 2024-07-01

**Authors:** Teodora McKenna, Dominic E McKenna, Vinson Fernandes, Marian Korda, Una Bradley

**Affiliations:** 1 Otolaryngology - Head and Neck Surgery, Queen's University Medical School, Belfast, GBR; 2 Otolaryngology - Head and Neck Surgery, Southern Health and Social Care Trust, Craigavon, GBR; 3 Endocrinology, Southern Health and Social Care Trust, Craigavon, GBR

**Keywords:** pathology, geriatrics, rare, orbital compartment syndrome, parathyroid adenoma, parathyroid carcinoma, hyperparathyroidism, hypercalcaemia, orbital cellulitis, bisphosphonates

## Abstract

Bisphosphonates are widely used for a number of metabolic bone conditions. Orbital inflammation is a very rare side effect of bisphosphonate therapy that can risk permanent visual loss. We describe the complex case and successful treatment of a 79-year-old man who developed orbital cellulitis following the use of intravenous pamidronate disodium for severe hypercalcaemia. The challenges regarding the diagnosis of parathyroid carcinoma are also discussed.

## Introduction

Initially synthesised as industrial anti-scaling compounds, the first study on the biological effects of bisphosphonates was published in 1969 [1]. They are now commonly prescribed for the management of osteoporosis, hypercalcaemia, and bone metastasis.

Bisphosphonates are classified into two groups, nitrogen-containing or non-nitrogen-containing, and vary in their efficacy and oral bioavailability. They are selectively absorbed by the mineral surface of the bone, disrupting osteoclastic bone resorption at an intracellular level [2].

Hypercalcemia is common, accounting for 0.6% of acute medical admissions [3]. Although primary hyperparathyroidism and malignancy are the most frequent underlying causes, there are numerous other causes including granulomatous conditions, drugs such as lithium, and familial hypocalciuric hypercalcaemia, necessitating a thorough clinical workup [3].

Along with intravenous hydration, bisphosphonates form the cornerstone of symptomatic moderate or severe hypercalcaemia treatment, with pamidronate disodium and zoledronic acid approved for this indication [4].

Orbital complications are very rarely reported with bisphosphonates, with only 42 cases of orbital inflammation documented worldwide [5]. Orbital cellulitis describes diffuse oedema of the orbital cavity posterior to the orbital septum, most commonly seen in children as a complication of ethmoid sinusitis. Urgent ophthalmology input is necessary as it risks increased intraorbital pressure, compressive optic neuropathy, and permanent blindness [6].

We present a complex case of a 79-year-old man who presented with severe hypercalcaemia arising from a parathyroid mass that produced excessively high levels of parathyroid hormone. This, along with the size of the mass, and the severity of his hypercalcaemia, raised the possibility of parathyroid carcinoma, a rare and notoriously difficult diagnosis to make on histological grounds [7]. Two days following the administration of pamidronate disodium for his hypercalcaemia, he developed acute onset periorbital and orbital cellulitis with loss of visual acuity and ocular motility that responded well to systemic corticosteroids.

## Case presentation

A 79-year-old man attended the geriatric rapid access clinic with a vague sense of ill health, reduced ambulation, and cognitive decline. His medical history was significant for a right inferior parathyroid adenoma excision for primary hyperparathyroidism in 2011, type 2 diabetes mellitus, and chronic kidney disease stage 3A (baseline creatinine of 160 umol/L). Unfortunately, he was lost to follow-up at a previous institution but was noted in retrospect to have mild persistent hypercalcaemia from 2014 to 2021 on routine blood testing, prior to the above presentation in the latter half of 2023.

Systematic questioning and examination elicited no specific features. Screening blood tests revealed a corrected calcium level of 3.48 mmol/L, a plasma parathyroid hormone (PTH) level of 1,099 ng/L, a serum creatinine of 222 umol/L, and an estimated glomerular filtration (eGFR) of 23 mL/min (Table 1). Vitamin D, urinary calcium, thyroid function, urine metanephrines, and vasculitis screen were all unremarkable.

**Table 1:** Blood test results

Laboratory parameter	On admission	Following bisphosphonate therapy	On discharge	Reference range
Plasma parathyroid hormone	692	1099	9	15-65 ng/L
Serum adjusted calcium	3.48	2.37	2.59	2.2-2.6 mmol/L
Serum albumin	41	32	36	35-50 g/L
Phosphate	0.76	0.81	1.42	0.8-1.5 mmol/L
Magnesium	0.67	0.71	0.61	0.7-1 mmol/L
Urinary calcium	6.55	-	-	2.5-7.5 mmol/24 h
Vitamin D	53	-	-	>50 nmol/L
Serum sodium	141	136	141	133-146 mmol/L
Serum potassium	4.4	4.1	4	3.5-5.3 mmol/L
Serum urea	11.4	13.1	17.9	2.5-7.8 mmol/L
Serum creatinine	205	282	235	59-104 umol/L
Estimated glomerular filtration rate (eGFR)	26	18	22	60-150 mL/min
C-reactive protein	5	72	3.5	0-5 mg/L
Total white cell count	6.7	7.8	7.9	4-10 x10^9^/L
Serum free T4	16.9	-	-	12-22 pmol/L
Serum TSH	1.58	-	-	0.27-4.20 mU/L
24h urine metanephrine output	791	-	-	<1289 nmol/24 h
Anti-myeloperoxidase antibody	<1	-	-	0.0-5.9 IU/mL
Anti-proteinase 3 antibody	<0.6	-	-	0.0-4.9 IU/mL
Serum angiotensin-converting enzyme	19.9	-	-	8-65 U/L

He had inpatient treatment with intravenous isotonic saline in addition to cinacalcet 30 mg twice daily. Despite nephrology and endocrinology input, his renal function continued to deteriorate (creatinine 335 umol/L, eGFR 14).

**Figure 1:**
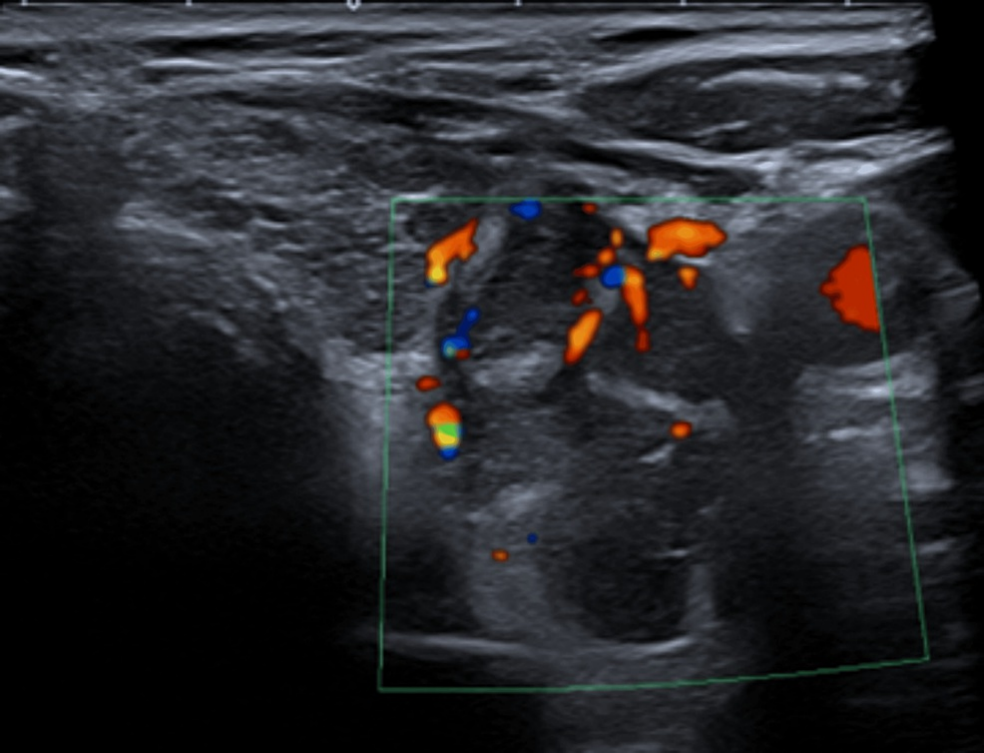
US parathyroid A 3 cm x 1.9 cm x 3.4 cm heterogeneous mass with increased vascularity adjacent to the lower pole of the left lobe.

Following an ear, nose, and throat doctor (ENT) consult, parathyroid gland ultrasonography showed a 3 cm x 1.9 cm x 3.4 cm mass adjacent to the lower pole of his left thyroid gland (Figure 1). It was heterogeneous and displayed increased vascularity. Its size and vascularity, along with the severity of his hypercalcaemia and hyperparathyroidism, raised concerns regarding parathyroid carcinoma.

**Figure 2:**
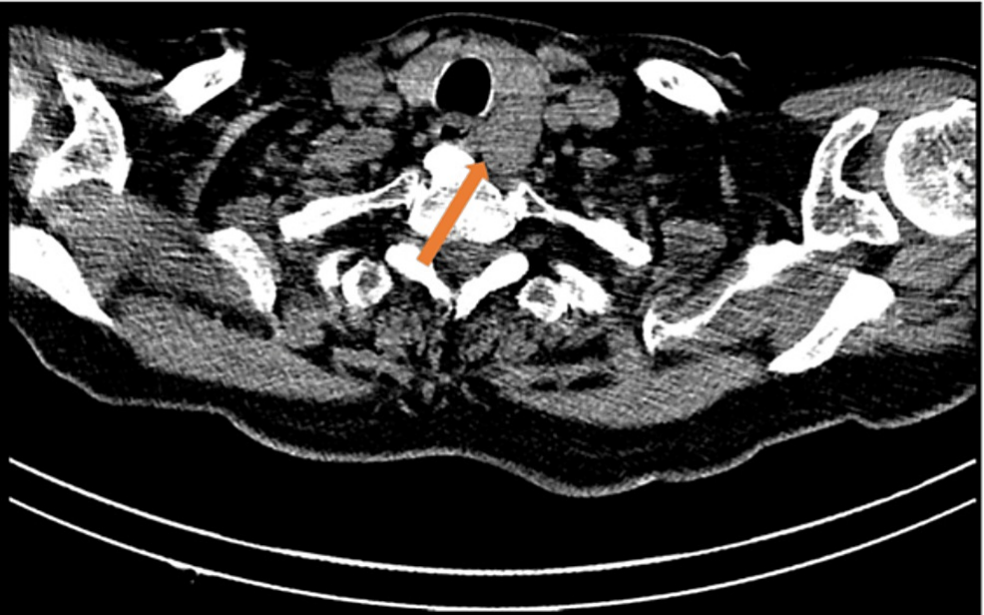
Nuclear medicine parathyroid SPECT – axial image Oval-shaped soft tissue mass posterior to the left thyroid lobe (orange arrow). SPECT - single photon emission computed tomography

**Figure 3:**
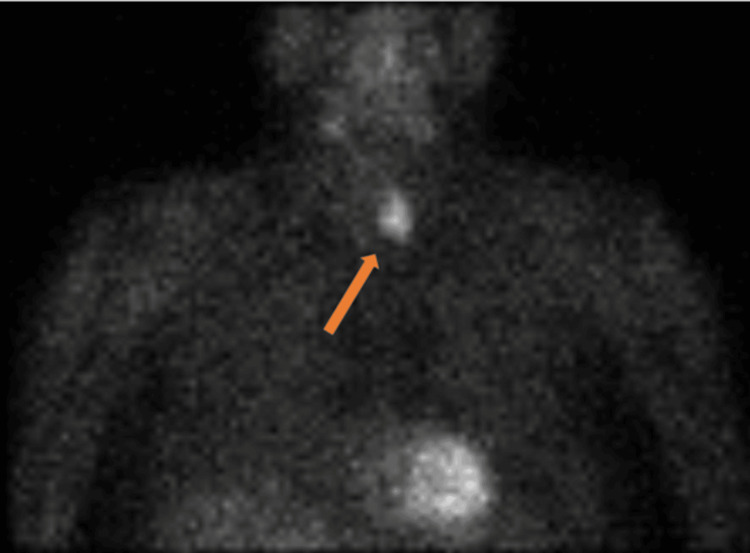
Nuclear medicine parathyroid SPECT – coronal uptake image Increased uptake within the soft tissue mass is evident (orange arrow). SPECT - single photon emission computed tomography

**Figure 4:**
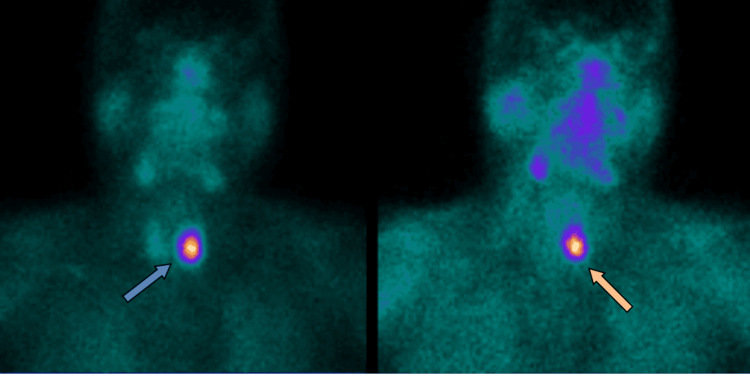
Nuclear medicine parathyroid MIBI – coronal image Persistent focal abnormal uptake in the region of the left thyroid lower pole. Blue arrow – scan after 10 minutes. Orange arrow – scan after 3 hours.

Subsequent nuclear medicine parathyroid technetium 99m sestamibi (Tc-MIBI) scintigraphy and single photon emission computed tomography (SPECT) scans showed persistent focal abnormal increased uptake within the parathyroid mass (Figures 2-4). His hypercalcaemia persisted despite an increase in cinacalcet dose to 90 mg four times daily. He then received a slow infusion of intravenous bisphosphonate at a reduced dose (pamidronate disodium 30 mg) in light of his renal function. This normalised his calcium levels within 72 hours.

**Figure 5:**
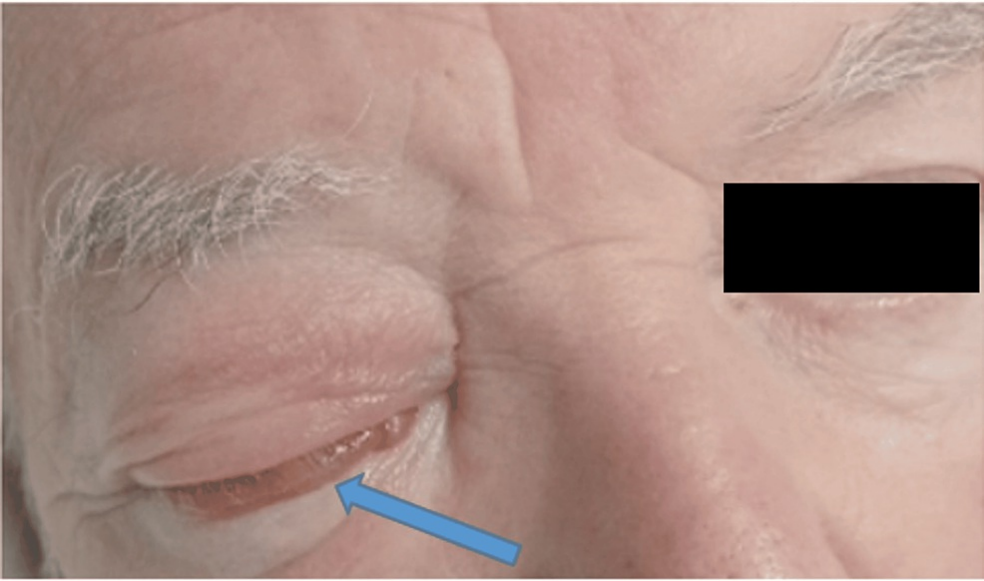
Periorbital cellulitis with chemosis (blue arrow) and inability to open the eye

Additionally, 48 hours following his intravenous bisphosphonate, he developed right orbital pain with upper and lower lid oedema (Figure 5). This progressed over five days despite intravenous flucloxacillin and topical 1% chloramphenicol ointment. His symptoms then acutely worsened with severe pain, chemosis, reduced visual acuity (6/24 from a baseline of 6/12), and ocular motion restriction. His colour vision and intraocular pressure were normal, with no relative afferent pupillary deficits and retina or corneal abnormalities. He had no associated neurological or rhinological symptoms, with unremarkable nasal endoscopy. His antibiotics were escalated to intravenous ceftriaxone and metronidazole although he remained apyrexic.

**Figure 6:**
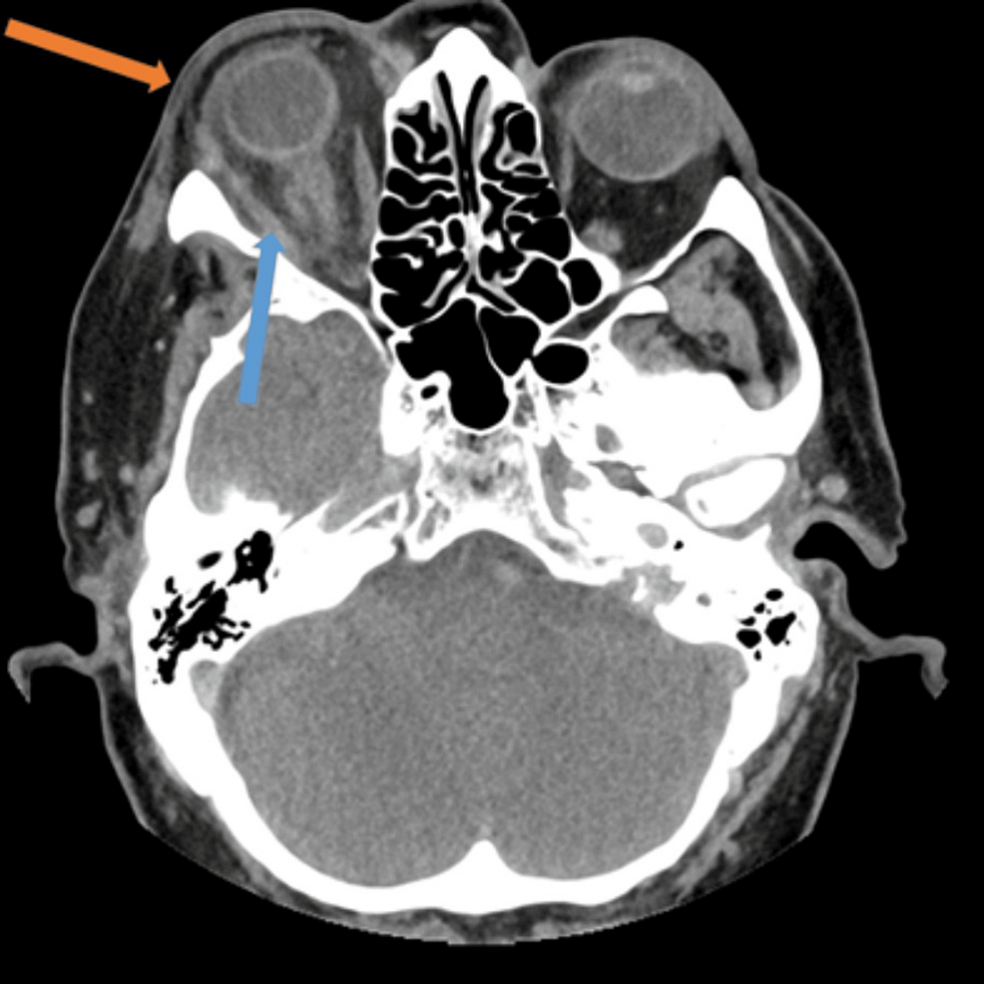
Contrast-enhanced CT orbits – axial image Periorbital soft tissue thickening (orange arrow) and oedematous extraocular muscles (blue arrow) with generalised orbital oedema. Note the presence of proptosis.

**Figure 7:**
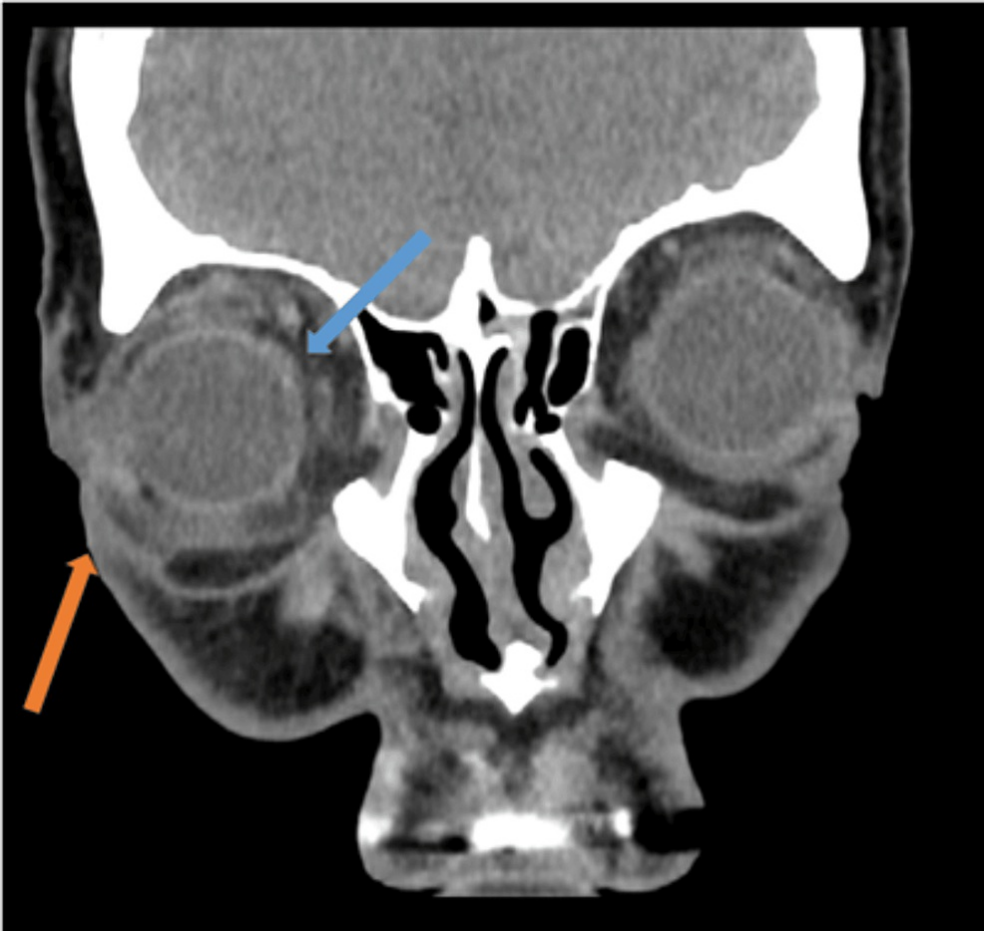
Contrast-enhanced CT orbits – coronal image Demonstrates orbital fat stranding (blue arrow) with significant periorbital oedema (orange arrow).

His total white cell count was within a normal range (5.30x10^9^/L) with moderate elevation in C-reactive protein (72 mg/L). A contrast-enhanced CT scan of his orbits showed evidence of orbital and periorbital cellulitis, with associated proptosis and inflammation of his extraocular muscles (Figures 6, 7).

Ophthalmology commenced him on 40 mg of oral prednisolone for 10 days, followed by a reducing dosage protocol, for suspected bisphosphonate-driven orbital inflammation. He also received topical dexamethasone 0.1% four times daily. The pain and swelling acutely improved, with the restoration of baseline visual acuity and ocular motion.

He later underwent an en-bloc resection of a firm fibrotic left parathyroid mass along with the adjacent closely adherent left thyroid lobe. A small 11 mm nodule was also removed from the neck at level six.

**Figure 8:**
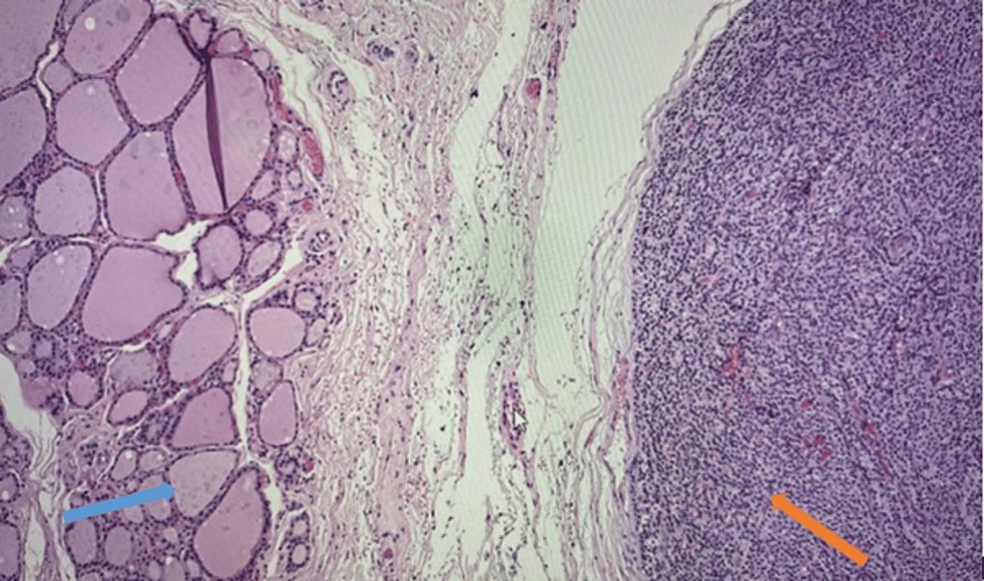
Lobulated tumor with pseudocapsule (orange arrow), composedly primarily of chief cells, abutting but without invasion of the adjacent normal thyroid parenchyma (blue arrow)

**Figure 9:**
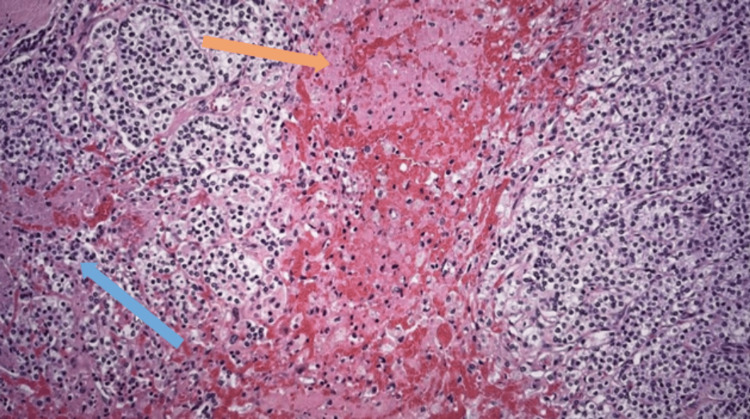
Parathyroid adenoma with abundant chief cells (blue arrow) and intervening fibrous septa (orange arrow)

Histology of the parathyroid mass showed a well-circumscribed lobulated tumour composed predominantly of chief cells, with a partial surrounding pseudocapsule (Figure 8). It showed no evidence of thyroid, blood vessel, and lymphatic or perineural invasion. Overall appearances were consistent with parathyroid adenoma (Figure 9). Histology of the level six lesion showed a cyst lined by parathyroid chief cells, consistent in the right clinical context with a cystic parathyroid adenoma.

His postoperative course was complicated by hungry bone syndrome requiring intravenous calcium infusions. His PTH and calcium levels normalised, and his creatinine improved to 233 umol/L. He remains under clinical follow-up with serial PTH and calcium measurements.

## Discussion

This complex and uncommon case has a number of widely generalisable learning points. Firstly, it underpins the importance of multidisciplinary work. During this patient's inpatient stay, he had input from geriatrics, acute medicine, nephrology, endocrinology, ENT, radiology, ophthalmology, and pathology for a number of significant concurrent issues. These included severe hypercalcaemia with acute kidney injury, a grossly elevated PTH level from a large parathyroid mass, acute orbital inflammation, and postoperative hungry bone syndrome.

Secondly, it adds to the body of literature on a very rare, poorly understood and vision-threatening complication of bisphosphonate therapy whilst serving to highlight a number of other uncommon but serious complications associated with this widely prescribed medication class [8].

Thirdly, it underscores the often-vague symptomatology of even severe hypercalcaemia. This patient also had a number of clinical features concerning the diagnosis of parathyroid carcinoma, a rare and notoriously difficult tumour to diagnose histologically [7].

Bisphosphonates are utilised for their ability to favourably alter bone metabolism in conditions, such as osteoporosis, hypercalcaemia, Paget’s disease, and bone metastasis. They bind to hydroxyapatite crystals, particularly at sites of high bone turnover, inhibiting osteoclast function and bone resorption. In nitrogen-containing bisphosphonates such as pamidronate disodium, this occurs due to inhibition of the intracellular enzyme farnesylpyrophosphate synthase (FPPS) [2]. Renal excretion limits their use in patients with creatinine clearance less than 30 mL/minute to life-threatening situations.

Although generally well tolerated, bisphosphonates can be associated with potentially severe side effects including osteonecrosis of the jaw and external auditory canal, as well as atypical femoral fractures and oesophageal ulceration [8-10].

Ocular complications of bisphosphonates are rare with conjunctivitis, anterior uveitis, episcleritis, and scleritis most frequently described [11]. Orbital inflammation as in our case is very rare, with only 42 cases reported worldwide [5]. This is a potentially devastating complication, risking irreversible optic neuropathy and blindness [6]. It is unilateral in 89% of cases, occurring within three days of intravenous pamidronate disodium and zoledronic acid, and typically between 15 and 21 days after oral alendronate [5].

Ocular inflammation following the administration of bisphosphonates may relate to the stimulation of gamma-delta T cells and the associated release of pro-inflammatory mediators such as tumour necrosis factor-alpha and interleukin-6. Due to their inhibition of farnesyl pyrophosphate, bisphosphonates indirectly stimulate gamma-delta T cells through the subsequent accumulation of the gamma-delta T-cell agonists dimethylallyl diphosphate and isopentenyl diphosphate [11,12]. This condition requires urgent ophthalmology input to exclude orbital compartment syndrome and commencement of systemic steroids to which it usually responds well (Table 2).

**Table 2:** Orbital compartment syndrome Orbital compartment syndrome is a life-threatening emergency. The presence of the above signs should prompt an urgent ophthalmology review [13].

	Orbital compartment syndrome
Clinical signs	Elevated intraocular pressure
	Decreased visual acuity
	Impaired colour vision
	Impaired extraocular movements
	Proptosis
	Chemosis
	Inability to open eye
	Relative afferent pupillary defect
	Intraocular vascular changes

Primary hyperparathyroidism (PHPT) is the most common cause of hypercalcaemia in the community and is associated with a broad range of symptoms, reflected in the classic mnemonic “stones, bones, groans and psychic moans” (Table 3). As our patient demonstrates, the presentation can be subtle, with hypercalcaemia often identified on routine testing in patients with nonspecific fatigue, bone pains, or cognitive issues [3].

**Table 3:** Symptoms of hyperparathyroidism Classic symptoms of hyperparathyroidism related to the associated hypercalcaemia [14].

Acronym	Presentation
Stones	Polyuria, polydipsia, dehydration, nephrocalcinosis
Bones	Bone and joint pain, osteoporotic fractures
Groans	Abdominal pain, anorexia, constipation
Psychic moans	Altered mental state, depression, fatigue

Parathyroidectomy is the only curative treatment, improving bone mineral density and quality of life whilst reducing the risk of renal stones, cognitive decline, and fractures [15]. About 2% of patients will have recurrent PHPT, defined as an elevation of previously normalised calcium and PTH six months after the initial operation [16]. The concern regarding parathyroid cancer, in this case, was due to the severity of his hypercalcemia, the size of the neck mass, and the extent of his PTH elevation (peak PTH level 17x higher than the upper reference range).

Parathyroid cancer is very rare, accounting for less than 1% of PHPT cases with an incidence of less than 1 per million per year [17]. The rule of threes should raise clinical suspicion, with a parathyroid mass >3 cm, severe hypercalcaemia >3 mmol/L, and PTH levels >3x the upper normal limit associated with the condition [17].

Diagnosis is challenging in the absence of metastatic disease, as histology is often not definitive [18]. The diagnosis may only be evident following later disease recurrence. The World Health Organisation (WHO) criteria require at least one vascular invasion, local invasion, metastasis, or recurrence for diagnosis [19]. Parathyroid cancer usually presents with severe hypercalcaemia from significantly raised PTH levels compared with the typically more mild elevations seen with benign parathyroid disease [17]. Treatment is with en bloc tumour resection along with the ipsilateral hemithyroid, with clear surgical margins the most important prognostic factor. The role of adjuvant therapy remains unclear. Recurrence rates of up to 50% have been reported although 10-year survival is about 70%, due to the slow-growing nature of the disease. The cause of mortality is usually uncontrolled hypercalcaemia [19].

In our case, the aforementioned biochemical abnormalities prompted oncological surgery for his parathyroid mass. The identification of parathyroid tissue in level six in this patient does raise the possibility of multi-centric parathyroid cancer [20]. Recurrence is usually heralded by a gradual increase in calcium and PTH levels, eventually leading to a hypercalcaemic crisis, hence the requirement for long-term follow-up [7].

## Conclusions

This case highlights the importance of coordinated multidisciplinary care in the approach to medical complexity. We have described the successful treatment of a rare potentially severe complication of BP treatment, highlighting the importance of prompt ophthalmological review in patients with BP complaining of new ocular symptoms.

We also discussed the complexities regarding the diagnosis of parathyroid cancer and the importance of the maintenance of a high index of suspicion, particularly given the prognostic implications of clear margins at the initial surgery.
